# Chronic lead encephalopathy in an adult: A case report and literature review

**DOI:** 10.1016/j.prdoa.2026.100450

**Published:** 2026-05-25

**Authors:** Tourby Rim, Sikkal Asmaa, Khattab Hajar, Haddouali Kamal, Bellakhdar Salma, El Moutawakil Bouchra, M.A. Rafai, El Otmani Hicham

**Affiliations:** Department of Neurology and Clinical Neurophysiology, Ibn Rochd University Hospital, Casablanca, Morocco

**Keywords:** Lead encephalopathy, Parkinsonism, Occupational exposure, Neurotoxicity, Movement disorders

## Abstract

We report the case of a 51-year-old man with lead encephalopathy due to chronic occupational exposure in fishing net manufacturing. Over three years, he developed cognitive decline, cerebellar ataxia, parkinsonism, and myoclonus. MRI showed bilateral hyperintensities T2/FLAIR sequences in deep brain structures, and elevated blood lead levels confirmed the diagnosis [2]. Chelation therapy led to slight improvement in motor symptoms, but cognitive deficits persisted [3]. This case highlights the importance of early recognition and management of lead neurotoxicity to prevent irreversible brain damage.

## 1 Introduction

Lead toxicity is a well-known occupational hazard, with encephalopathy representing its most severe neurological manifestation. Although more common in children, chronic exposure in adults can also lead to significant cognitive and neurological impairment. This case report emphasizes the importance of considering lead poisoning in the differential diagnosis of unexplained neurological disorders.

## 2 Case description

**Fig. 1:**
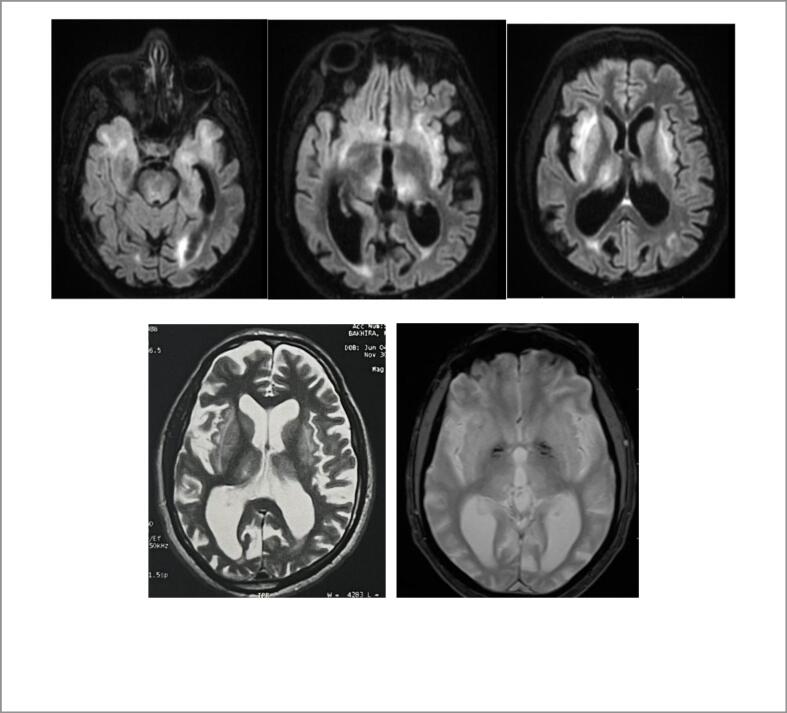
Pre-treatment brain MRI (T2-FLAIR) shows lesions in the insular-temporo-occipital lobes, and lesions in the caudate nucleus, thalamus and the pons. T2* reveals calcifications in the lentiform nucleus.

A 53-year-old man, employed as a fishing net manufacturer since age 12, presented with a three-year history of progressive neurological symptoms, including unsteady gait, cognitive decline, bradykinesia, and movement disorders. Neurological examination revealed ataxia, severe symmetric parkinsonism, positive and negative myoclonus in action in the upper limbs, hyperreflexia, and a dysexecutive syndrome with apraxia and attentional deficits (MMSE 16/30). A dark blue line along the gums was noted (See Fig. 1).

**Fig. 2:**
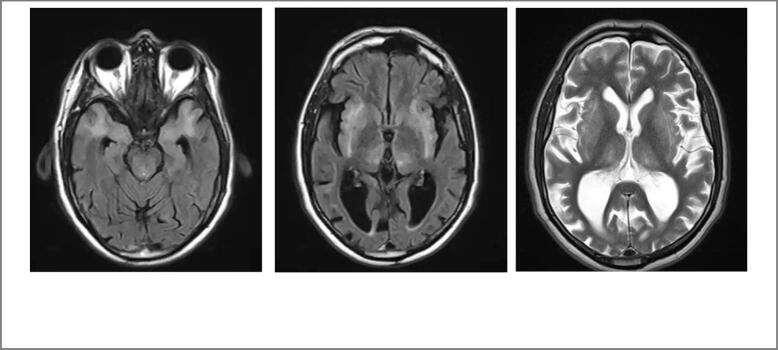
Post-treatment MRI shows a slight resolution of lead deposits.

No ocular, bulbar, sensory, autonomic, or gastrointestinal symptoms were reported. Brain MRI demonstrated bilateral symmetrical hyperintensities affecting the thalamus, caudate nucleus, external capsule, pons, and temporo-occipital/insular lobes on T2-FLAIR images, without diffusion restriction or contrast enhancement. T2* sequences showed hypointensities in the lentiform nucleus suggestive of calcifications And see Fig. 2 after this : And follow-up MRI showed partial resolution of lesions.

EEG revealed generalized slowing (6 Hz). Cerebrospinal fluid analysis showed elevated protein (1.28 g/L) with normal glucose and cell count. Serum copper, ceruloplasmin, and autoimmune encephalitis antibodies were normal. Routine blood tests were unremarkable .

Given his occupational exposure and gingival discoloration, lead poisoning was suspected. Blood lead level was markedly elevated at 899 µg/L (normal < 85 µg/L), confirming lead encephalopathy.

The patient was treated with oral dimercaptosuccinic acid (DMSA) 10 mg/kg × 3/day during 5 days, resulting in gradual improvement of motor symptoms over two months, although cognitive impairment persisted. Blood lead levels decreased to 713 µg/L, and follow-up MRI showed partial resolution of lesions.

## 3 Discussion

This case highlights the importance of considering chronic lead exposure in patients with unexplained neuropsychiatric or movement disorders, particularly in occupational settings. Common sources include battery, paint, ammunition, cosmetic industries, mining, smelting, plumbing, and herbal medicines. In this case, exposure occurred through fishing sinker manufacturing, an uncommon but relevant source.

Lead enters the body through inhalation, ingestion, or skin breaches, accumulating in blood, soft tissues, and bone. It crosses the blood–brain barrier by mimicking calcium ions, disrupting synaptic transmission and leading to neuronal apoptosis and cerebral edema. Lead neurotoxicity induces parkinsonism through oxidative stress, mitochondrial dysfunction, and disruption of dopaminergic and noradrenergic systems in the basal ganglia, rather than classical nigral neurodegeneration. Cerebellar Purkinje cell vulnerability accounts for ataxia, while cortical hyperexcitability underlies the myoclonus.

Previous studies report that affected workers are often middle-aged with prolonged exposure [1]. Our patient had exposure since childhood and developed symptoms progressively over three years, including cognitive decline, cerebellar dysfunction, and parkinsonian features. Myoclonus and akinetic-rigid syndrome indicated basal ganglia involvement.

MRI findings in this case, including bilateral T2/FLAIR hyperintensities in deep brain structures and T2* evidence of calcifications, are characteristic of lead encephalopathy. The mechanism of intracranial calcification remains unclear.

CSF findings showed elevated protein with normal glucose and cell count, which is consistent but nonspecific [2]. Blood lead measurement remains the diagnostic gold standard. While normal levels are <10 µg/dL, encephalopathy typically occurs above 100 µg/dL, though lower levels may suffice.

Lead encephalopathy may mimic autoimmune or infectious encephalitis, such as anti-NMDA receptor encephalitis or herpes simplex meningoencephalitis, due to overlapping imaging findings. This emphasizes the importance of occupational history and toxicological screening.

Treatment involves removal from exposure and chelation therapy (DMSA, succimer, or D-penicillamine). In this patient, DMSA reduced blood lead levels and improved motor symptoms, but cognitive deficits persisted, suggesting irreversible cortical damage. This aligns with previous reports showing better recovery of motor function than cognition.

Follow-up MRI demonstrated partial resolution of lesions, indicating that chelation can reduce toxicity but may not fully reverse brain damage. Persistent abnormalities in the thalamus and basal ganglia likely explain ongoing cognitive impairment, highlighting the chronic and potentially irreversible nature of lead neurotoxicity [3], [4], [5].

## 4 Conclusion

Lead encephalopathy should be considered in patients presenting with unexplained neuropsychiatric or movement disorders, especially in the context of occupational or environmental exposure. Its ability to mimic other neurological conditions underscores the need for thorough evaluation, including detailed exposure history and blood lead measurement.

Early diagnosis and treatment are essential to limit neurological damage, although prognosis remains guarded in chronic cases. MRI plays a key role in diagnosis and follow-up. While chelation therapy can improve motor symptoms, cognitive deficits may persist, emphasizing the importance of prevention and early intervention in at-risk populations.

## Declaration of competing interest

The authors declare that they have no known competing financial interests or personal relationships that could have appeared to influence the work reported in this paper.
